# Questioning Lead Standards: Even Low Levels Shave Points off IQ

**Published:** 2005-07

**Authors:** Tina Adler

The maximum blood lead concentration deemed acceptable for children has declined over the years, from 60 micrograms per deciliter (μg/dL) in 1970 to the present-day level of 10 μg/dL, first established in 1991. In the last several years, however, researchers have begun to suspect that even lower concentrations may impair cognition. Now a reevaluation of data from seven international longitudinal studies involving 1,333 children confirms this suspicion **[*EHP* 113:894–899]**.

The studies—conducted in Boston, Cincinnati, Cleveland, Rochester (New York), Port Pirie (Australia), Mexico City, and Yugoslavia—originally looked at children known to be at risk for lead poisoning, such as those living near lead smelters or in deprived urban settings. Therefore, the majority of the participants had blood lead levels far higher than the averages currently being reported in the developed world. The mean blood lead concentration for the entire group peaked at 17.8 μg/dL at age 2.5 years, and declined to 9.4 μg/dL between ages 5 and 7. Only 18% of the children had maximal blood lead levels of less than 10 μg/dL, and 8% had maximal blood lead levels of less than 7.5 μg/dL.

Most of the children took IQ tests when they were between almost 5 and 7 years of age; the Boston children were tested at age 10. The current team calculated, across the seven studies, how much of the difference in IQ scores was related to lead alone by controlling for other factors that influence IQ scores, including child birth weight, birth order, prenatal exposure to tobacco smoke and alcohol, and mother’s IQ.

On a population basis, an increase in blood lead level from 2.4 to 10 μg/dL at the time of testing was associated with a decrease of 3.9 IQ points. At lower blood lead levels, a small increase in blood lead made a bigger difference in IQ than the same size increase did at higher concentrations. A blood lead level of 20 μg/dL was associated with scoring about 1.9 points lower on tests of IQ compared with a blood lead level of 10 μg/dL. The difference in IQ shrank to 1.1 points when comparing a blood lead level of 20 μg/dL with a concentration of 30 μg/dL.

To determine if the data from one particular study drove the final results, the team removed the findings for one site at a time and recalculated the results. It became clear that no single study was driving the results of the pooled analysis.

Consistent with a study published in the May 2005 issue of *EHP*, blood lead level at the time of IQ testing was generally a stronger predictor of effects on IQ than was—as previously believed—blood lead level at age 2. The individual-level effect on IQ is difficult to determine, however, and may depend in part on the child’s social environment.

In the United States, about 2–3% of children have a blood lead concentration above 10 μg/dL, but in some cities, such as Rochester, 1 in 5 children have elevated blood lead. These new findings, along with those from previous human and animal studies, point to the importance of eliminating nonessential uses of lead and lowering allowable levels of lead in air emissions, house dust, soil, water, and consumer products.

## Figures and Tables

**Figure:**
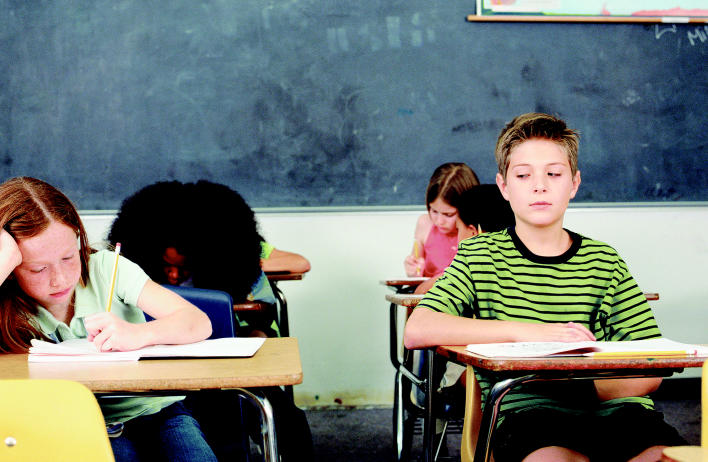
**The test of time.** More studies are confirming the surprise finding that blood lead concentration at the time of IQ testing, not peak level, is a better predictor of IQ effects.

